# Application of Thermography and Adversarial Reconstruction Anomaly Detection in Power Cast-Resin Transformer

**DOI:** 10.3390/s22041565

**Published:** 2022-02-17

**Authors:** Kuo-Hao Fanchiang, Cheng-Chien Kuo

**Affiliations:** Department of Electrical Engineering, National Taiwan University of Science and Technology, Taipei 106335, Taiwan; d10707007@mail.ntust.edu.tw

**Keywords:** anomaly detection, variation autoencoder, generative adversarial networks, defect detection, infrared thermography, power transformers, winding insulation

## Abstract

Dry-type power transformers play a critical role in the power system. Detecting various overheating faults in the running state of the power transformer is necessary to avoid the collapse of the power system. In this paper, we propose a novel deep variational autoencoder-based anomaly detection method to recognize the overheating position in the operation of the dry-type transformer. Firstly, the thermal images of the transformer are acquired by the thermal camera and collected for training and testing datasets. Next, the variational autoencoder-based generative adversarial networks are trained to generate the normal images with different running conditions from heavy to light loading. Through the pixel-wise cosine difference between original and reconstructed images, the residual images with faulty features are obtained. Finally, we evaluate the trained model and anomaly detection method on normal and abnormal testing images to demonstrate the effeteness and performance of the proposed work. The results show that our method effectively improves the anomaly accuracy, AUROC, F1-scores and average precision, which is more effective than other anomaly detection methods. The proposed method is simple, lightweight and has less storage size. It reveals great advantages for practical applications.

## 1. Introduction

Power transformers play a critical role in the power system. A dry-type transformer refers to the electric transformer in which the core and winding are cast together with epoxy resin, instead of being immersed in oil. The dry-type power transformer is usually installed in indoor applications due to the advantages of a lower maintenance workload, high operating efficiency, compact size and low noise. However, in view of the rapid growth of power demand, the stability of the dry-type transformer is gradually becoming a noteworthy problem, which directly affects the reliability of the entire power grid [1,2,3].

There are different ways of insulating and dissipating heat between dry-type and oil-immersed transformers. Oil-immersed transformers are insulated by insulating oil, and the heat generated by the coil is transferred to the radiator of the transformer via the insulating oil inside the transformer for heat dissipation. Dry-type transformers are generally insulated with resin, cooled by natural air or by fans. Compared to the liquid-filled transformer, one of the disadvantages of dry-type is that, due to lack of oil for insulation or cooling, they require sufficient cooling capacity [4]. There are still some shortcomings in the operation of the dry-type transformer system, especially material defects in the process of manufacturing, that it is unrecoverable once damaged and so on [5]. Common failure modes of the dry-type transformer fall into insulation failure in coils, conduct failure, mechanical damage, etc., which are prone to overheating and pose a serious threat to the safe operation of the transformer. As reported by Islam et al. [6], the winding insulation failure is responsible for almost 45% of transformer failures, one of which is the inter-turn fault, which is the most common. The inter-turn fault occurs because of the abnormality of the winding insulation due to aging and later extends to the inter-layer discharge. Insulation capacity has become the most critical indicator affecting transformer performance and reliability [7]. The temperature of the dry-type transformer is of great importance to reflect the transformer operating condition. Higher temperatures will accelerate the insulation aging of the equipment and shorten the lifetime of the equipment. The key part of a dry-type transformer is insulation, which will inevitably cause failure due to thermal aging [8]. Most of the transformer failures are accompanied by overheating before being damaged [9]. Therefore, if the heat trend of the fault point can be detected early, accidents can be prevented and reduced in time.

Generally, all kinds of transformer failures have a huge impact on the power grid. So far, many monitoring techniques and detection methods have been proposed to diagnose the faults for the transformer [10,11,12,13]. Existing transformer fault detection technologies mainly include the sweep frequency response analysis (FRA) method [10], excitation current test [11], partial discharge (PD) analysis [12] and vibration signals [13]. The FRA, excitation current, vibration signals and PD are the most common methods in practice and have some disadvantages such as external noise, offline monitoring, incorrect placement etc. in the implementation. Mostly, these methods present major shortcomings for real-time measurements due to high electromagnetic interference, being time-consuming and requiring professional knowledge, which is an obstacle for long-term monitoring and accurate identification of the transformer’s fault. [14].

Infrared thermography (IRT) is the technology that converts invisible heat energy into visible thermal images. The advantages of IRT are that its characteristics are non-invasive and non-contact, as well as its mobility [15]. Several papers made use of IRT to determine the transformer failure. Laib et al. [16] suggested using a support vector machine (SVM) to find the region of interest (ROI) by pre-processing the image and then applying the well-trained SVM classifier to recognize the data. In [17], they employed to perform infrared image segmentation, preprocessing and feature extraction and then used the naive Bayes classifier to classify the rotating machinery failure. Recently, deep learning has caused a major impact in the field of computer vision [18]. Affected by deep learning algorithms, many researchers have developed several methods for thermal image detection using deep learning.

Anomaly detection is a common application of machine learning algorithms, which mainly finds out the unexpected or anomalous data or patterns in various unsupervised learning problems. Anomaly detection methods usually use available normal data to extract, characterize and model the patterns, and then develop reasonable anomaly detectors to discover new or abnormal patterns in the newly observed data [19,20,21]. Due to being scarce and difficult to gather enough abnormal samples, unsupervised learning methods are more effective to use in most practical application scenarios [22,23]. Surveys on anomaly detection in power transformers were conducted as follows. Mitiche et al. [24] utilized a long short-term memory autoencoder network for anomaly detection in the current magnitude and phase angle from three bushing taps. This method models the normal current and phase measurements of the bushing and infers any point when these measurements change based on the mean absolute error metric evaluation. Liang et al. [25] and Tang et al. [26] collected the original data distribution of power transformer-dissolved-gas in oil during normal operation. Then, the normal data are clustered by using the K-means clustering method, and the abnormal data are distinguished from the normal one according to the distance between both.

In the past few decades, work has been carried out on image anomaly detection techniques, and we focus on several methods related to the proposed method. Bergmann et al. [27] propose to use the convolution autoencoder architecture based on a per-pixel L2 loss or a structural similarity index (SSIM) loss function. This approach can detect the difference between local image regions, considering the luminance, contrast and structural information instead of simply comparing single pixel values. AnoGAN [28] is the model that uses the GAN technique for anomaly detection. The basic idea of this work is to train the GAN model through normal images so that the resulting GAN will generate normal images based on noise. By comparing the generated image and the abnormal image, the noise of the input GAN is updated through their differences so as to obtain the normal image corresponding to the abnormal image, so that even the location of the abnormality can be found. f-AnoGAN [29] is most closely related to AnoGAN, which still requires the mapping from image to latent space during detection. Different from that, AnoGAN has the disadvantage of iterative computational inefficiency in detection time; the f-AnoGAN technique replaces this iterative procedure with a learned mapping from image to latent space, dramatically improving speed. GANomaly is proposed by Akçay et al. [30] to introduce an adversarial network. The objective of the GANomaly model is not only to minimize the difference between the original and generated images but also to minimize the difference within their latent vector representations jointly. Although GANomaly has the advantage of reducing the calculation load and the good performance of anomaly detection, the quality of the generated image of GANomaly is not enough to handle all tasks. Vu [31] has proposed one-class classification and anomaly detection in computer vision using adversarial autoencoders. This approach made use of the benefits of an autoencoder architecture and adversarial training and took the discriminator reconstruction error as an anomaly score for detecting whether the input image is anomalous. Lai et al. [32] introduce a robust subspace recovery layer (RSR layer) within an autoencoder for anomaly detection, which enforces an outliers-robust linear structure in the embedding obtained by the encoder. This structure can define a subspace where only inlier data lie in while outlier data are out. Wang et al. [33] adopt VQ-VAE and PixelSnail, respectively, as the reconstruction model and autoregressive model to get the latent representation of normal samples and estimate the probability model of the latent representation. They detect the abnormality by comparing the L2 distance between the restored image and the defective image. Tang et al. [34] developed a dual auto-encoder generative adversarial network (DAGAN) with skip-connection to perform the task of anomaly detection. This study combines three loss functions as the training objective to verify the reconstruction ability and training stability. The residual score of the test image calculated by L2 distance is superior to some thresholds, which is judged as abnormal.

In this paper, we develop a novel deep variational autoencoder-based anomaly detection technique for cast-resin power transformers. First, we employed an IRT image-captured system to monitor the operation of the power transformer and handle training and testing data preparation. Then, we trained a variational autoencoder-based generative adversarial network on only defect-free images with different running conditions from heavy to light loading, as discussed in Section 3. The objective of the model is to learn features of defect-free samples during training. After that, we evaluated the trained model and anomaly detection method on normal and abnormal testing images to demonstrate the effeteness and performance of the proposed work. From the evaluation, we performed deep anomaly detection experiments based on the cast-resin transformer IRT images (CT-IRT) dataset proposed by Fanchiang et al. [35]. There are various dry-type transformer defection types per class in this dataset. Additionally, we compared it with other deep anomaly detection approaches based on unsupervised learning. In summary, the main contributions of this study are as follows:

1. We propose an unsupervised anomaly detection method system based on IRT image recognition. Without a large number of labeled defect images, the system can monitor the operation of the transformer online and find the outlier situation early.
2. Compared with other existing methods, the proposed method leads to significantly finer reconstructed images and achieves good results in terms of anomaly accuracy, AUROC, F1-scores and average precision.
3. The proposed method also has the advantages of fewer weights and less storage space. In addition, ablation studies are conducted to verify the effectiveness of the combination of the loss function.

The rest of this paper is organized as follows. Section 2 briefly introduces the theory and algorithms of the variational autoencoder (VAE), generative adversarial networks (GANs). Section 3 describes in detail the proposed approach. Detailed experiments and performance results are carried out in Section 4. Finally, the conclusions are drawn in Section 5.

## 2. Theoretical Background

### 2.1. Variational Autoencoder Generator

The autoencoder (AE) performs efficient feature extraction and feature representation on high-dimensional data based on an unsupervised learning method. The AE framework contains two networks: encoding process EN and decoding process DE [36]. The basic idea of AE is that after throwing a piece of input through a neural network, it will get a compressed representation of the same input data. First, the “input training data $X_{input}=\left[x_{1},\ldots x_{n}\right]$ $(x_{i}\in R^{n}$)” is put into the EN networks. The EN networks compress it into an intermediate vector represented by low-dimensional code $h_{EN}=\left[h_{1},\ldots \;h_{m}\right]$ $(h_{i}\in R^{m}$). The transformation process of the encoder can be described as follows:

(1) $$h_{EN}=EN\left(X_{input}\right)=Act_{EN}\left(W_{En}^{k}\times X_{input}+b_{EN}^{k}\right)$$

where $W_{EN}^{k}$ is the k-th weight matrix of the encoder function with the dimension of n×m and $b_{EN}^{k}$ is the k-th bias vector of the encoder function with the dimension m. $Act_{EN}$ is the non-linear conversion of the encoder. This intermediate vector code can be used as an input feature vector. After that, the output of the EN function is sent into DE networks, which produces output $\hat{x}$. The transformation process of the decoder can be described as follows:

(2) $$\;\hat{x}=EN\left(h_{EN}\right)\;=Act_{DE}\left(W_{DE}^{k}\times h_{EN}+b_{DE}^{k}\right)$$

where $W_{DE}^{k}$ is the k-th weight matrix of the decoder function with the dimension of m×n and $b_{DE}^{k}$ is the k-th bias vector of the decoder function with the dimension n. $Act_{DE}$ is the non-linear conversion of the decoder. Rectified linear unit (ReLU) is selected as the activation function of $Act_{EN}$ and $Act_{DE}$, as seen in the following equation:

(3) $$Act_{EN},Act_{DE}=\left\{\begin{array}{c} 0\;,\;a\leq 0\\ a\;,\;a>0 \end{array}\right.$$

Although AE requires that the original image and the reconstructed image be as similar as possible, this process cannot control the distribution of the latent space. The high degree of freedom of the autoencoder makes the encoding and decoding not lose information, which will lead to serious overfitting.

Kingma and Welling proposed the variational autoencoder (VAE) [37] to control the latent space to produce a reconstructed image that is more similar to the original image. The network architecture of VAE is similar to that of AE. In VAE, the way to master the distribution of the latent variables z is to first force to project the probability distribution of input images $P\left(x\right)$ into some type of prior probability distribution $P\left(z\right)$, or equivalently $P\left(z|x\right)$. In this paper, we use the standard normal distribution with mean zero and variance one, $N\left(\mu ,{\;\sigma }^{2}\right)$ as $P\left(z|x\right)$. The representation of the latent space for each image can be described as follows:

(4) $$z_{i}=\mu_{i}+\sigma_{i}\cdot \varepsilon$$

where $\mu_{i}$ and $\sigma_{i}$ are the mean and standard deviation vectors of the encoded features from the *i*th input image and ε is the sample from the standard normal distribution. The objective function of VAE is expressed as Equation (5), which has two terms, $L_{REC}$ and $L_{KLD}$.

(5) $$L_{VAE}=L_{REC}+L_{KLD}$$

The reconstruction loss function $L_{REC}$ in Equation (6) is the L1 distance (Manhattan distance) error, which is the summation of the differences between the corresponding elements of the output and the input. The parameters of the encoder and decoder are adjusted to minimize the error between the input and the final output. If the output $\hat{x}$ is very similar to the input x, it is believed that the intermediate vector code h has a certain relationship with the input x (that can be mapped).

(6) $$L_{REC}=\sum L\left(x,\hat{x}\right)=\sum \|x-\hat{x}{\|}_{L1}$$

The Kullback–Liebler divergence loss $L_{KLD}$ in Equation (7) is the similarity measure between the encoder’s distribution $Q_{\theta }\left(z|x\right)$ and $P\left(z\right)$, which adjusts the weight θ of the encoder by encouraging the approximate posterior $Q_{\theta }\left(z|x\right)$ to match the prior $P\left(z|x\right)$. This also means that we want to regularize $Q_{\theta }\left(z|x\right)$ to be as close as possible to the standard normal distribution.

(7) $$L_{KLD}=KL_{D}[Q_{\theta }\left(z|x\right)||P\left(z|x\right)]=E_{Q_{\theta }\left(z|x\right)}[\log Q_{\theta }\left(z|x\right)-\log P\left(z|x\right)]$$

where $KL_{D}$ means the operation of Kullback–Liebler divergence.

### 2.2. Generative Adversarial Networks

The model structure of the generative adversarial network (GAN) is also an unsupervised learning method, which learns by making two neural networks confront each other. The GAN consists of a generative network G and a discriminative network D. The G tries to generate an image, while the D network decides whether the generated image is real or fake. D and G play the following two-player minimax game with value function [38]. The loss function for the generator $L_{G}$ and the discriminator $L_{D}\;$of GAN can be depicted as follows:

(8) $$L_{G}=E\left[\log(D\left(G\left(z\right)\right)\right]\;L_{D}=E\left[\log\left(D\left(x\right)\right)\right]+E\left[\log(1-D\left(G\left(z\right)\right)\right]$$

GANomaly was presented by Akcay et al. [30], which is the model composed of the autoencoder generator networks and the discriminator networks. The objective function of the generator ($L_{GANomaly}$) is given by the following equation:

(9) $$L_{GANomaly}=W_{adv}L_{adv}+W_{con}L_{con}+W_{enc}L_{enc}$$

where $W_{adv}$,$W_{con}$ and $W_{enc}$ are weighted parameters for the use to change the influence of $L_{adv}$, $\;L_{con}$ and $L_{enc}$ on the entire loss function. The feature matching error ($L_{adv}$) in Equation (10) is calculated between the feature of the original image x and the generated image $\hat{x}$, which is the way to optimize the image feature layer. The function $f\left(.\right)$ means the intermediate layer of the discriminator network. The reconstruction error loss ($L_{con}$) in Equation (11) is used to reduce the gap between the original image x and the reconstructed image $\hat{x}$ at the pixel level. Lastly, the encoder loss ($L_{enc}$) in Equation (12) can decrease the difference between the latent representation of the input z and the encoding feature of the generated image $\hat{z}$.

(10) $$L_{adv}=\|f\left(x\right)-f\left(\hat{x}\right){\|}_{L2}$$

(11) $$\;L_{con}=\|x-\hat{x}{\|}_{L1}\;$$

(12) $$\;L_{enc}=\|z-\hat{z}{\|}_{L2}\;$$

Wasserstein GAN (WGAN) presented by Arjovsky et al. [39] improves the training performance of GAN via the use of Wasserstein distance. This training technique is proposed to perform faster convergence and stability of GAN training. Wasserstein distance not only indicates the distance between two distributions but also represents how to transform from one distribution to another. The value function of Wasserstein distance is expressed as follows:

(13) $$W\left(p_{data},p_{g}\right)=\underset{D\in 1-Lipschitz}{\max}\left\{E_{x\sim p_{data}}\left[D\left(x\right)\right]-E_{\hat{x}\sim p_{g}}\left[D\left(\hat{x}\right)\right]\right\}$$

where $p_{data}$ is the original input data and $p_{g}$ is the generated data. The concept of the Lipschitz function is to confirm whether the function $f\left(.\right)$ is still smooth in the interval between $x_{1}$ and $x_{2}$, because if this function changes greatly, it will not satisfy the following conditions:

(14) $$\left\|f\left(x_{1}\right)-f\left(x_{2}\right)\|\leq K\|x_{1}-x_{2}\right\|$$

The advantage of using Wasserstein distance as a loss function is that even if there is no intersection between $p_{data}$ and $p_{g}$, the Wasserstein distance can still be measured by the distance between both probability distributions, so the generator can learn better step by step during the training process.

Inspired by GANomaly and WGAN, the proposed variational autoencoder generator (VAG) model applied in the full-time online fault detecting in this paper takes the reconstruction error, Kullback–Liebler divergence, the adversarial feature error and Wasserstein distance as the loss function with the corresponding weighting parameters to adjust each loss.

## 3. The Proposed Anomaly Detection Approach

**Figure 1:**
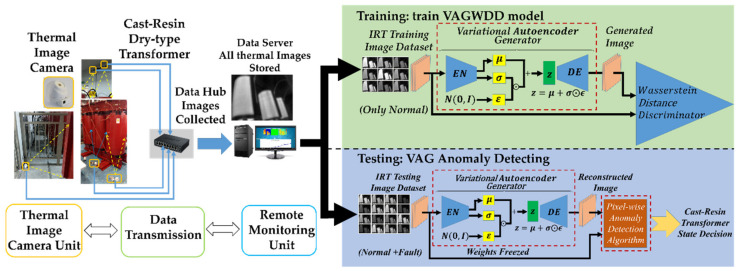
Flowchart of the proposed cast-resin transformer anomaly detection system based on the Variational Autoencoder Generator and the Wasserstein Distance Discriminator (VAGWDD) model.

The proposed framework, as seen in Figure 1, is divided into three stages: image acquisition and dataset establishment, offline training model and online anomaly detecting. First, the IRT images for the normal and fault condition were captured by the proposed monitoring system. These images are collected into training and test data sets. Next is the offline training phase, which focuses on the adversarial training of the variational autoencoder generator (VAG) model. The VAG model is trained to generate images with normal states. Finally, in the test phase, the residual image is obtained by computing the absolute value of the pixel-wise cosine distance between the real image and the generated image, which directly shows the result after the inference analysis: normal or abnormal (and determine what kind of fault).

### 3.1. Image Acquisition

The dataset is collected by capturing images of the dry-type transformer in a substation by using the fixed thermal imaging cameras as shown on the left in Figure 1. In this paper, the single thermal camera is installed in such a way that it can capture the total view of the transformer, which can take pictures as the dataset in this study. The infrared camera system is composed of a fixed-focus lens assembly, a long-wave infrared (LWIR) microbolometer sensor array and signal-processing electronics. The array format of the thermal camera is 80 × 60 pixels available, which can measure object temperature up to 120 °C. Thermal images acquired by a thermal camera are scaled to the resolution of 120 × 160 pixels via the application of the software. The field of view for diagonal and horizontal are 63.5° and 50° wide angles, respectively. The thermal sensitivity has an accuracy of about 0.05 °C. The proposed method is mainly based on the image comparison between the real operating state and the regenerated normal state. Compared with other IRT-based methods, we do not need to measure the difference of allowable temperature rise, but only concentrate attention on calculating the image difference between the above-mentioned states. In this work, the proposed method focuses on identifying anomalous types and locations.

The daily average load rate is calculated based on the size of the load and the length of usage time. The loading change rate of the transformer is 20–65%. The average loading rate is 34.2%, and the daily average temperature of the ambient temperature is 30 °C–35 °C. The monitoring system captures the running normal IRT images of the transformer under different load conditions from light to heavy with a thermal camera and sends these images to the remote server through the internet. Each IRT image is normalized and converted into the 120 × 160 × 3 grayscale image, which is regarded as the input dimension of the proposed model. The voltage level of the observed transformer is 24 kV. The maximum ambient temperature, temperature-rise limitation and maximum permissible temperature are 40 °C, 100 K and 155 °C, respectively. The standard capacity, primary voltage and second voltage are 1000 KVA, 24 kV and 380 V, respectively.

### 3.2. Offline Training Model

The training is divided into two steps as shown in Figure 1 (green box), where the training IRT image can be seen. At the first step, we focus on training the model that can reconstruct the normal image through generative adversarial learning to fully catch the features of the normal image. At the second step, the classification model is trained for detecting the nine transformer conditions. To improve the robustness of the model, gaussian noise is added to each image before training.

The VAGWDD model parameters are initialized. $L_{d\_loss}$ and $L_{g\_loss}$ are the WDD loss and the VAG loss, respectively. The WDD model is first updated several times via $L_{d\_loss}$ to let the WDD distinguish the difference between the real image and the generated image. Next, the WDD is fixed to train VAG via $L_{g\_loss}$ once. Training WDD via minimizing $L_{d\_loss}\;$in Equation (15) is exactly like the Wasserstein distance $W\left(p_{data},p_{g}\right)$ divergence in Equation (13) [39].

(15) $$L_{d\_loss}=-\left[WDD\left(x\right)\right]+\left[WDD\left(\hat{x}\right)\right]$$

where x and $\hat{x}$ are the real images and the generated images, respectively.

The generator loss function $L_{G\_loss}$ in Equation (16) proposed in this study refers to VAE [37] and GANomaly [30] architecture. The loss combination of image reconstruction and Kullback–Liebler divergence, called the$\;L_{VAE}$, the WDD image feature loss $L_{WDD-fea}$ and the Wasserstein loss $\;L_{was}$ are regarded as the loss function of the proposal with corresponding weights,$\;W_{VAE}$, $W_{WDD-fea}$ and $W_{was}$.

(16) $$L_{G\_loss}=W_{VAE}L_{VAE}+W_{WDD-fea}L_{WDD-fea}+W_{was}L_{was}$$

The loss combination of image reconstruction and Kullback–Liebler divergence, $L_{VAE}$, in Equation (17), reveals the difference between the generated image $\hat{x}$ and the original image x and forces the generation of a latent vector with the standard normal distribution.

(17) $$L_{VAE}=L_{REC}+L_{KLD}$$

In this paper, we use L1 loss formulation as the image reconstruction loss $L_{REC}$ in Equation (18) to minimize the error that is the sum of the all the absolute differences between both images. In order to make the generated image more similar to the original image, the reconstruction error must be as small as possible.

(18) $$L_{REC}=\|x-\hat{x}{\|}_{1}=\overset{n}{\underset{i=1}{\sum }}\left|x-\hat{x}\right|$$

The Kullback–Liebler divergence $L_{KLD}$ in Equation (19) is the measure of the distance between z which comes from a Gaussian with mean σ, variance μ and the standard normal distribution. The encoder $Q_{\theta }\left(z|x\right)$ produces an approximate posterior distribution $Q\left(z|x\right)$, and is parametrized with weights θ. The decoder $P_{\varphi }\left(x|z\right)$ grants a likelihood distribution $P\left(x|z\right)$, and is parametrized with weights φ. In this paper, the encoded distributions are chosen to be normal so that the encoder can be trained to return the mean and the variance value that describes these Gaussians. The reason why the input is encoded as a Gaussian distribution is that it is an easy way to express latent space regularization. The distributions returned by the encoder are enforced to be close to a standard normal distribution. The Kullback–Leibler divergence between two Gaussian distributions has a closed form that can be directly expressed in terms of the means and the variance values of the two distributions. The value of $KL_{D}$ is down to zero, which means that two probability distributions are as close as possible and make the encoder $Q_{\theta }\left(z|x\right)$ learn the latent variables with multivariate normal distribution. Due to the re-parameterization trick, the variational approximation $Q_{\theta }\left(z|x\right)$ of the proposed VAG model is a Gaussian distribution. The KL-Divergence $L_{KLD}$ is thus given by:

(19) $$L_{KLD}=KL_{D}[Q_{\theta }\left(z|x\right)||P\left(z|x\right)]=\frac{1}{2}\overset{K}{\underset{k=1}{\sum }}\left(1+\ln({\left(\sigma_{k}\right)}^{2}\right)-{\left(\mu_{k}\right)}^{2}-{\left(\sigma_{k}\right)}^{2})$$

To identify the WDD image feature loss $L_{WDD-fea}$ in Equation (20), the generated image and the original image are sent to the function $f_{WDD}$ that outputs an immediate layer of the WDD model. The L2 loss formulation is adopted to minimize the error which is the sum of all the squared differences between both the images.

(20) $$L_{WDD-fea}=\|f_{WDD}\left(x\right)-f_{WDD}\left(\hat{x}\right){\|}_{2}=\overset{n}{\underset{i=1}{\sum }}{\left(f_{WDD}\left(x\right)-f_{WDD}\left(\hat{x}\right)\right)}^{2}$$

The Wasserstein loss ($L_{was}$) in Equation (21) is a way to train the generator model steadily to approach the distribution of the IRT image with a normal state. The properties of the Wasserstein loss are continuous and differentiable. Therefore, the training process is more stable and less sensitive to model architecture. The larger the scores for generated images the WDD outputs, the smaller the VAG loss becomes.

(21) $$L_{was}=-\left[WDD\left(\hat{x}\right)\right]=-\left[\mathrm{WDD}\left(\mathrm{VA}G\left(x\right)\right)\right]$$

### 3.3. Online Anomaly Detecting

Anomaly detection based on the reconstruction methods usually depends on the computation of the difference between the input and the generated data to check whether the status is normal or abnormal. In order to perform anomaly detection tasks, the detection process needs to be designed, as shown in Figure 1 (blue box). First, input the IRT images x to be tested into the VAG model. After the VAG model reconstructs the images $\hat{x}$ corresponding to the normal state of the input images, use the pixel-wise anomaly detection algorithm to calculate the pixel-level anomaly residual score $Ars\left(i,j\right)$ between x and $\hat{x}$. In this paper, we leverage the cosine distance [40] as the anomaly residual score which is defined in Equation (22), to calculate the difference for each pixel of both images.

(22) $$Ars\left(i,j\right)=1-\frac{x\left(i,j\right)\;\cdot \;\hat{x}\left(i,j\right)}{\|x\left(i,j\right){\|}_{2}\|\hat{x}\left(i,j\right){\|}_{2}}=1-\frac{\sum x\left(i,j\right)\;\cdot \;\hat{x}\left(i,j\right)}{\sqrt{\sum x{\left(i,j\right)}^{2}}\sqrt{\sum \hat{x}{\left(i,j\right)}^{2}}}$$

The higher anomaly residual score represents more likely anomalies. Due to the model trained by only normal samples, the anomaly residual score of the input images with the normal state will be lower than the one with the abnormal state. By tuning the appropriate threshold, when the pixel-level anomaly residual score of the test image is higher than or equal to the test threshold, in other words, $Ars\left(i,j\right)\;$≥ α, the operation state of the cast-resin transformer is abnormal. The testing process is to directly shuffle the IRT images testing set and randomly input them into the well-trained model.

## 4. Experiments

In this section, we first describe the composition of the dataset and some training settings, other methods used to compare our model and the implementation details of our proposed framework. Secondly, the detection performance of our proposed model compared to other state-of-the-art unsupervised anomaly detection methods. Thirdly, we evaluate ablation experiments to explore the effectiveness of each additional component of the proposed work.

The proposed IRT-based anomaly detection method is trained to get the capability to interpret the thermal images coming from the thermal camera and find out the operation outlier state for a dry-type transformer. The training and testing processes are coded in Python 3.6 using TensorFlow Keras and run on Windows 10 64-bit operating system with GTX 1650 GPU.

### 4.1. Experiments Setup

#### 4.1.1. Dataset Establishment

In order to verify the effectiveness of detecting anomalies in IRT images of the cast-resin transformer, we refer and leverage the CT-IRT dataset proposed by Fanchiang et al. [34] for evaluation. The dataset has a total of 1322 samples. Of these samples, 300 images are of the normal condition and are only used to train the variational autoencoder generator (VAG) model in the training step. The rest of the total samples are used for the testing. The normal state of the testing dataset is marked as “F0”. The label F1, F2 and F3, respectively, means the inter-turn short-circuit of the R, S and T phases. The label F4, F5 and F6, respectively, means the connection overheating of phases R, S and T. The label F7 and F8, respectively, means the overheating of the wires in the S and T phases. Detailed statistics of the dataset in this work is in Table 1.

#### 4.1.2. Defect Description

Most transformer failures are accompanied by the symptom of overheating. In this paper, the proposed method focuses on detecting the overheating location of the cast-resin transformer. Local overheating is regarded as the early warning of the failure which probably brings about burning. If the fault phenomenon is detected early, the transformer can be disconnected in time for maintenance to avoid catastrophe losses.

Common abnormal overheating phenomena of dry-type transformers include short circuits between local layers of transformer windings or between turns of transformer windings, loose internal contacts, increased contact resistance, etc. The inter-turn fault is due to the deterioration of the high-voltage side winding insulation. Poor insulation will cause discharge between layers, which will further cause winding short circuits and temperature rises. The contact overheating is at the junction of the primary side and the secondary side. Loose connections, overloads, unbalanced loads, etc., may cause the contacts to overheat. The cables connected to the transformer will generate heat due to unstable load. These faults can be observed with obvious hot spots from IRT images. The inter-turn short-circuit is caused by the winding insulation deterioration fault of dry-type transformers. In the early stage of the fault, due to the deterioration of the high-voltage winding insulation layer, interlayer discharge damage is caused, which results in the winding becoming short-circuited, the current increasing and the temperature going up. The connection overheating abnormality is located at the connection between the primary side and the secondary side, and the contact surface is usually locked with screws to transmit the current. The irregular phenomena, such as loose connection, overload, unbalanced load due to construction or excitation vibration may lead to overheating. Overheating of wires is often found in the connecting cables to the transformer. The cause of overheating usually stems from overload, load imbalance, load failure and other reasons. At the high voltage coils of cast-resin transformers, local overheating can often occur because of inter-turn short circuits arising from the demolition of the solid insulation by partial discharge.

#### 4.1.3. Baselines

We match the proposed method against the following state-of-the-art unsupervised anomaly detection methods:

- AE-L2 [27] and AE-SSIM [27]: We try to compare the convolutional autoencoder model in terms of the capability of anomaly detection. We train an autoencoder on only normal datasets. So, when input data that have different features from the normal dataset are fed to the model, the corresponding pixel-wise reconstruction error will increase via L2 distance and SSIM, respectively. The network architecture is composed of nine convolutional layers for the encoder and nine deconvolutional layers for the decoder. Leaky rectified linear units (ReLUs) [41] with slope 0.2 are implemented as activation functions after each layer, except for the output layers. The output size is 128 × 128 × 3.
- f-AnoGAN [29]: f-AnoGAN is a model based on GAN for anomaly detection. This method needs to train two components: the WGAN [39] and the encoder, and it tries to generate the nearest normal image for the test image with the WGAN generator trained only on the normal data. A characteristic of this model is that two adversarial networks (Generator and Discriminator) and the encoder are trained separately. Besides, an anomaly score is calculated by both a discriminator features residual error and an image reconstruction error. The output size is 64 × 64 × 3.
- GANomaly [30]: GANomaly is an encoder–decoder structured GAN which utilizes adversarial, contextual and encoder losses. The method trains only on normal data. In inference time, it re-generates normal images that contain the features of the input image whether the input image is a normal or abnormal sample. GANomaly predicts whether the new image from test data is normal or not by the following formula which is the L1 distance between the encoded original image by the first encoder and the encoded generated image by the second encoder.
- ADAE [31]: Vu et al. has advanced the semi-supervised methods, called the Adversarial Dual Autoencoders (ADAE) model, which has two autoencoders as generators and discriminators to stabilize during the process of training. The method uses the reconstruction error and conventional adversarial loss of an autoencoder-based model for anomaly detection. The output size is 64 × 64 × 3.
- RSRAE [32]: Lai et al. designed a special regularizer into the embedding of the encoder to enforce an anomaly subspace recovery layer with the energy-based penalty, which is called the Robust Subspace Recovery Autoencoder (RSRAE). RSRAE trains a model using the normal data and is applied to detect outliers at the testing. The anomaly score is computed by cosine similarity metrics, which means the higher reconstruction errors are viewed as outliers.

#### 4.1.4. Network Setup

**Figure 2:**
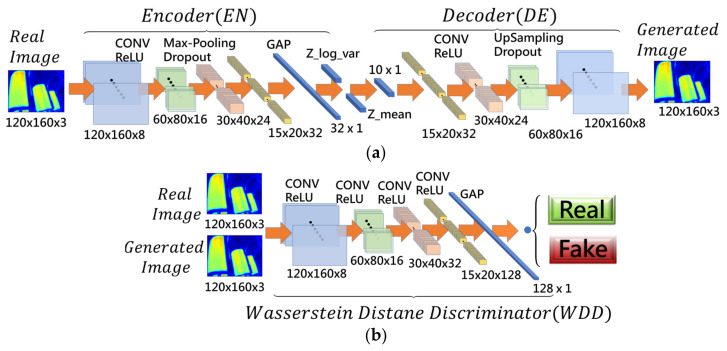
The detailed structure of the proposed VAGWDD model. (**a**) Variational Autoencoder Generator (VAG) networks; (**b**) Wasserstein Distance Discriminator (WDD) networks.

The reconstruction model architecture is composed of the VAG networks and the WDD networks, together called the VAGWDD model, which are shown in Figure 2. The network structure of VAG can be divided into two subnetworks: encoder and decoder.

The first subnetwork is the encoder which contains four convolutional layers; the filter size of the first convolutional layer is 5 × 5 and the filter size of the remaining three layers is 3 × 3. After each convolution layer, the batch normalization layer and ReLU are executed. At the end of the encoder, global average pooling is performed to converge the dimension to 32, which are followed by two linear fully connected layers for mean and variance. The output of the encoder is given by the sampling layer.

The second subnetwork is the decoder which contains four transposed convolutional layers with a filter size of 3 × 3. The input of the decoder is the sampling layer. After each transposed convolutional layer, ReLU is executed. The decoder output is a 120 × 160 × 3 regenerated image.

The combination of the first subnet and the second subnet forms the VAG network, as shown in Figure 2a. The encoder (EN) first is fed into the input IRT images x, where $x\in R^{w\times h\times c},w\times h\times c=120\times 160\times 3$. After the use of four convolution layers with batch-norm and ReLU activation, EN makes the input x compress into a vector z, where $x\in R^{d}$. z has a smaller dimension, including the feature representation of x. The decoder (DE) utilizes four convolutional transpose layers, which upscales the vector z to reconstruct the image x as $\hat{x}$. In summary, the VAG reconstructs image $\hat{x}$ via $\hat{x}=DE\left(z\right)$, where $z=EN\left(x\right)=\mu_{k}+\sigma_{k}\cdot \varepsilon$, in Equation (4).

For the WDD model, we used four convolutional layers with 3 × 3 kernel and 2 × 2 stride ReLU activation and one global average pooling layer. Lastly, the output of the WDD model was formed by one linear unit. After each convolutional layer, the batch normalization layer and ReLU are executed. The fourth convolutional layer extracts the features (15 × 20 × 128) to compute the error between the features of the generated image and of the original image. The smaller the error, the closer the features are. Immediately after the fourth layer, the global average pooling is performed to converge the dimension to 128. Finally, the single fully connected layer, which enables the sigmoid function, is used to identify the real image from the generated image.

#### 4.1.5. Training Details

**Figure 3:**
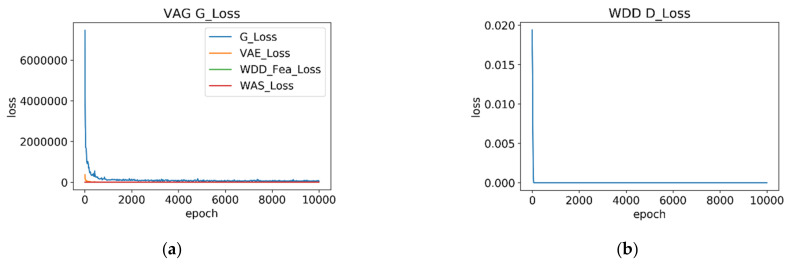
The training loss curve at every 20 epochs over 10,000 epochs of the VAG model with WDD. (**a**) the VAG G_loss curve; (**b**) the WDD D_Loss curve.

In the first stage of training, the VAG image reconstruction model is trained to learn and extract features from 300 normal state samples. The first steps involve obtaining a batch of 32 original images, and then the discriminator is trained. After that, the discriminator parameters are frozen, and then the generator is trained. The generator loss function in Equation (15) has weights of $W_{VAE}$ = 20, $W_{WDD-fea}$ = 1 and $W_{was}$ = 5. As shown in Figure 3, after 10,000 epochs training, the generator loss and discriminator loss are recorded every 20 epochs. As can be seen from Figure 3, after only 1000 epochs, the loss values of the VAG ($L_{G\_loss}$) and the WDD ($L_{D\_loss}$) have become stable. The weight of VAG has begun to gradually converge.

To assess the quality of the reconstructed image, several methods of the image quality assessment (IQA) for evaluation were used. The most common evaluation methods were PSNR (Peak Signal-to-Noise Ratio) and SSIM (Structural Similarity) [42]. PSNR is defined by the maximum pixel value (denoted as L) and the mean square error (MSE) between the images. Given the real image I and the reconstructed image $\hat{I}$ with N pixels, the MSE (mean squared error) of the two images I and $\hat{I}$ were calculated and transferred to dB domain, the higher the PSNR (dB unit). The quality of the generated image is better. In this paper, we let L equal to 255 in general cases using 8-bit representations.

**Figure 4:**
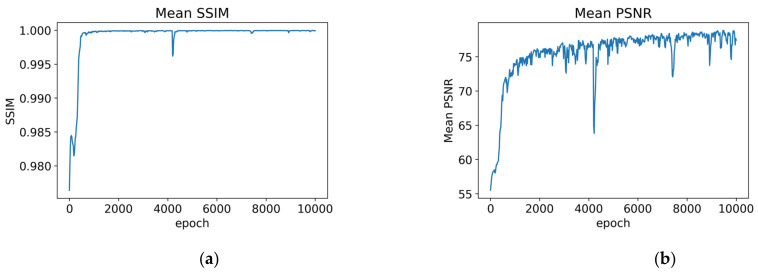
The curve of the VAG model training for every 20 epochs over 10,000 epochs. (**a**) Mean_SSIM; (**b**) Mean_PSNR.

Figure 4 shows the changes in PSNR and SSIM for 10,000 epochs training. It shows that PSNR and SSIM can reach over 75 dB and 0.9999 at the initial training stage (around 1660th epochs). As the number of training increases, although it cannot stabilize, the PSNR and SSIM values still have the trend to reach the relatively optimal value. In this article, the criteria are set that the quality of the reconstructed image is at least average PSNR = 77 dB, SSIM = 0.9999 or better when the model is trained at a certain epoch. This paper takes the best 10 records from 500 records of PSNR and SSIM, as shown in Table 2. Mean_SSIM and Mean_PSNR respectively indicate the average value of SSIM and PSNR after calculating for 300 images. Following the criteria, the No.1 model (at epoch = 7240) in Table 1 is the best-trained model to generate normal images in the second training step.

### 4.2. Evaluation Metrics

To evaluate the effectiveness and performance of our proposed work and other methods, we take the binary classification accuracy, the area under the receiver operating characteristic curve (ROC-AUC), F1-score and average precision (AP) for one (anomalous) vs. anomaly-free as our evaluation metrics.

We define true positive (TP), true negative (TN), false positive (FP) and false negative (FN), respectively, as the number of correctly classified abnormal images, the number of correctly classified normal images, the number of incorrectly classified abnormal images and the incorrectly classified normal images.

For the binary classification scenario, we calculate the accuracy of correctly classified images for outlier and inlier test images.

(23) $$\mathrm{Accuracy}=\frac{TP+TN}{TP+FP+TN+FN}$$

The most used metrics when evaluating anomaly detection solutions are F1 score, Precision and Recall. F1 score is the harmonic mean of precision and recall and gives a better measure of the incorrectly classified cases than the accuracy metric.

(24) $$\mathrm{Precision}=\frac{TP}{TP+FP}$$

(25) $$\mathrm{Recall}=\frac{TP}{TP+FN}$$

(26) $$F1\;\mathrm{score}={\left(\frac{{\mathrm{Recall}}^{-1}+{\mathrm{Precision}}^{-1}}{2}\right)}^{-1}$$

The area under the curve of the receiver operating characteristics (AUROC) is one of the most important measurements for evaluating the detection ability of binary detection. The receiver operating characteristics (ROC) is a probability curve and AUROC represents the degree or measure of separability. The ROC curve is plotted with TPR against the FPR, where TPR is on the *y*-axis and FPR is on the *x*-axis. The higher the AUROC is, the better the model can distinguish the input images between abnormal and normal.

The AP is a measurement for summarizing the precision-recall curve into a single value representing the weighted mean of precisions achieved at each threshold. The AP is calculated by using a loop that goes through all precisions and recalls. The difference between the current and next recalls is computed and then multiplied by the current precision.

(27) $$\mathrm{AP}=\overset{n-1}{\underset{i=0}{\sum }}\left[\mathrm{Recall}\left(i\right)-\mathrm{Recall}\left(i+1\right)\right]\times \mathrm{Precisions}\left(i\right)$$

where $\mathrm{Recall}\left(n\right)=0$ and $\mathrm{Precisions}\left(n\right)=1$. n is the number of the threshold.

### 4.3. Comparison Results

#### 4.3.1. Comparison against Other Methods

In this work, we respectively show the results calculated based on four different evaluation indicators and compare them with other recent related methods to assess the achievement of the proposed method. Label F0 means the normal testing images. Labels from F1 to F8 represent the abnormal testing images with different fault locations. Table 3 presents the outstanding ability of the proposed approach in the image-level classification task, which is based on the average accuracy of correctly classified images for abnormality and normality. Our proposed approach yields better binary classification results in all categories, with improvements ranging from 0.1% to 57.3%. Our method is not as good as AE-L2 [27] in the F6 vs. F0 category due to some anomalies like small spots that make the distinction more difficult, but the other accuracy results are still better than the existing method.

As summarized in Table 4, our results at AUROC were better than the second AE-L2 [27] with a 14.9% improvement. AUROC is classification-threshold-invariant, which describes the entire two-dimensional area underneath the entire ROC curve from (0,0) to (1,1). It measures the quality of the model’s predictions irrespective of what classification threshold is chosen. Due to the excessive difference between abnormal and normal scores and the good reconstruction ability of our proposed model, there is no better that can compete with ours.

We further compute the F1 Score to evaluate the anomaly detection performance of the model. As depicted in Table 5, the average F1 score is 0.944, demonstrating that the proposed model is capable of correctly differentiating between anomalous and normal images. The objective of the F1 score is to combine the precision and recall metrics into a single metric. The F1 score gives equal weight to precision and recall. If the model has a higher F1 score, that means the value of precision and recall are also higher, and vice versa. The meaning of the model with a medium F1 score is that one of precision and recall is low and the other is high.

Average Precision (AP) points out the performance of the model, which can correctly distinguish all positive examples without unexpectedly labeling other negative examples as positive. AP is estimated for the area under the curve that measures the balance between precision and recall at different decision thresholds. As shown in Table 6, our approach outperforms different common methods with an average AP of 0.941. We think the proposed model gives relatively good results with the mean value 0.145 higher than AE-L2. The AP score is high, which means the model can correctly handle positives.

In order to further compare the amount of memory required of the deep learning model during the testing process, the number of parameters and size for each model evaluated in this paper is given in Table 7. The number of parameters and the weight storage of the proposed method are 0.12 million and 0.55 MB, respectively. Our method has fewer weights and less storage space than other methods.

**Figure 5:**
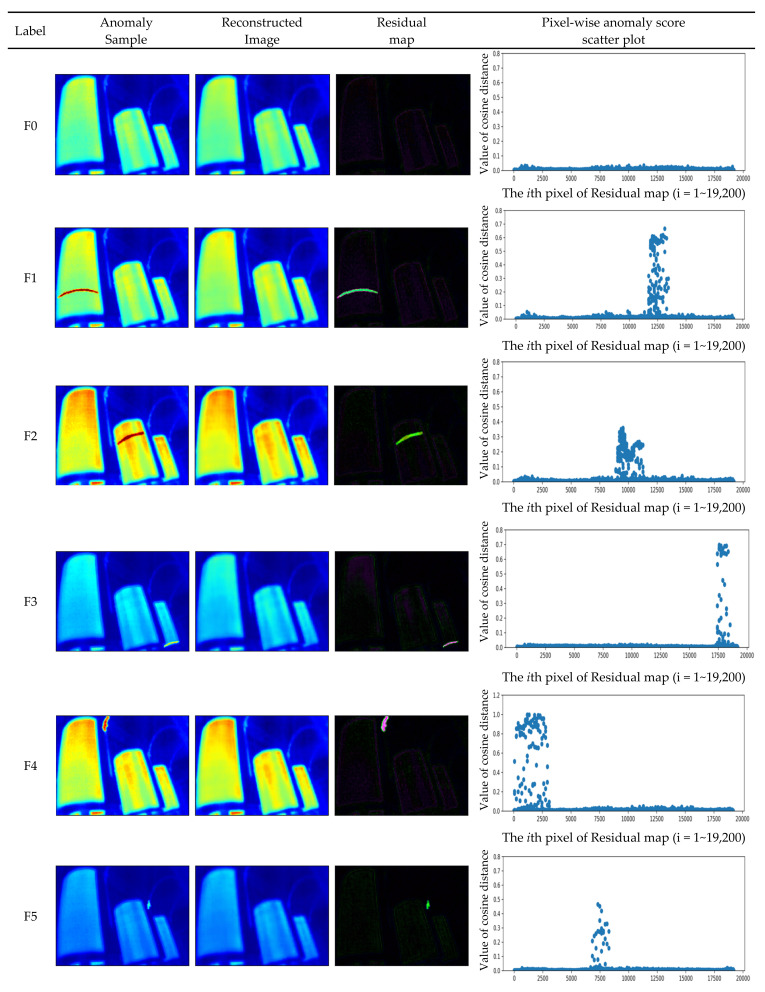

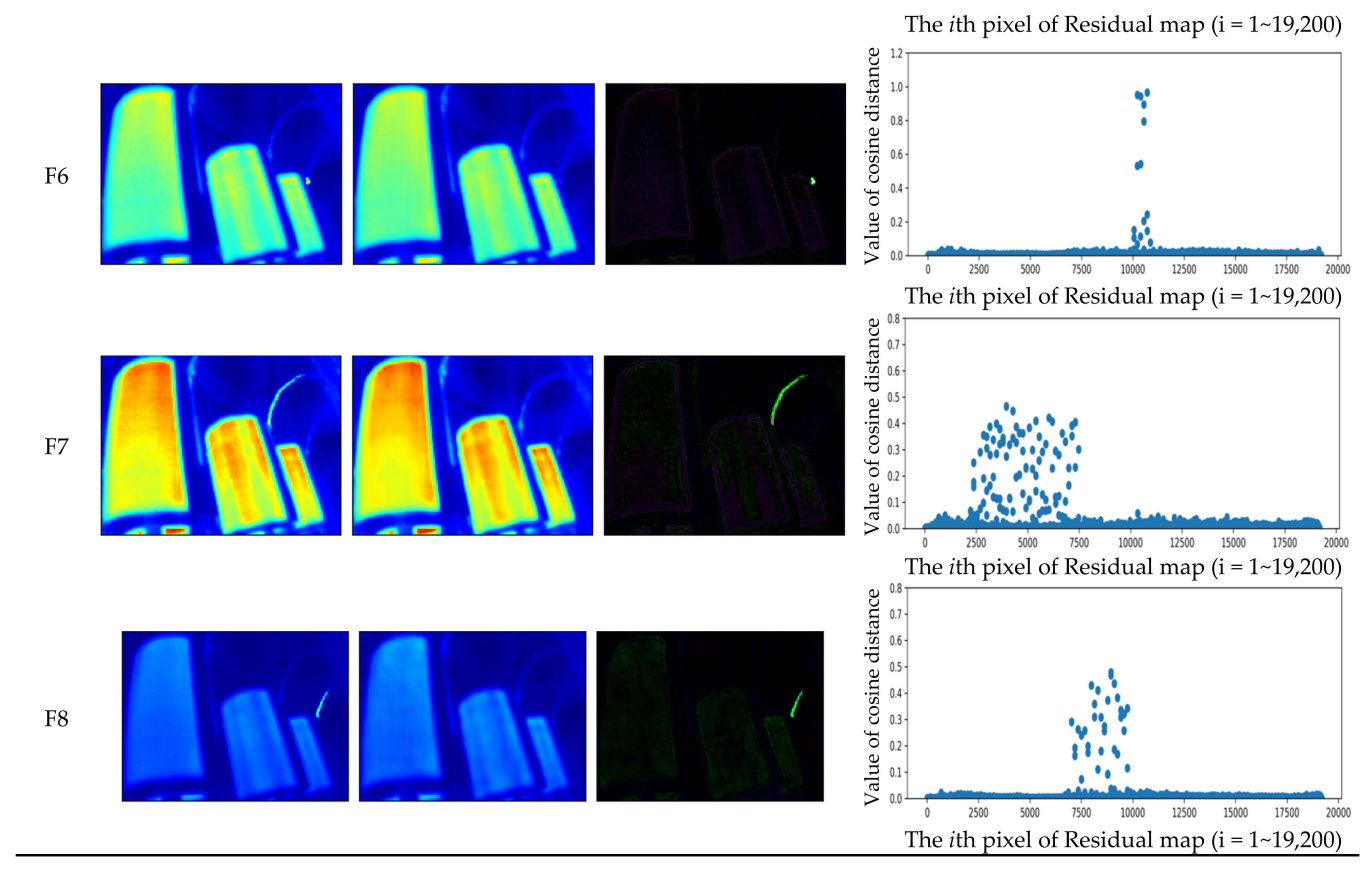
Qualitative results of the CT-IRT dataset generated by the proposed method VAG model.

Figure 5 shows all kinds of anomaly samples, the reconstructed images generated by the proposed method after inputting the CT-IRT dataset, the residual map calculated by the difference between the anomaly samples and the reconstructed images and the pixel-wise anomaly score computed by cosine distance. It is worth noting that the proposed method can identify which sample is abnormal and which one is normal. In Figure 5, the pixel-level anomaly score scatter plot in the residual map can clearly show the location and number of pixels of the fault, which is very important for power transformer detection. By calculating the cosine distance for each pixel, we can get the result that the difference in value between the abnormal and normal pixel is at least 100 times more than the one between the normal and normal pixel, so it is not too difficult to identify the fault.

#### 4.3.2. Comparison against Other Distance Metric

Distance metrics are a very critical approach for unsupervised anomaly detection. These distance metrics are generally used to calculate the distance error between images to measure the similarity [43,44]. When the calculation of the distance error results in a small quantity, it means that both images are close to each other; when it is big, they are different. In this work, we present separately the results calculated by the three different distance metrics. For a fair comparison, we use the proposed model well-trained by 300 normal training images for this testing in order to show the fact that the effective distance metric improves the performance of our approach.

- The Euclidean distance (Euclidean): the L2-norm of the difference, a special case of the Minkowski distance with *p* = 2. It is the natural distance in a geometric interpretation.
- The squared Euclidean distance (Sqeuclidean): like the Euclidean distance metric but does not take the square root. As a result, classification with the Euclidean squared distance metric is faster than classification with the regular Euclidean distance.
- The Manhattan distance (Manhattan): the L1-norm of the difference, a special case of the Minkowski distance with *p* = 1 and equivalent to the sum of absolute difference.

Table 8 shows that the cosine distance adopted by the proposed method outperforms other distance metrics in the accuracy of correctly classified normal or anomalous images on the CT-IRT dataset. The result shows the fact that the proposed method outperforms all the listed distance metrics in AUROC, F1 scores and AP on the CT-IRT dataset.

### 4.4. Ablation Experiment

**Figure 6:**
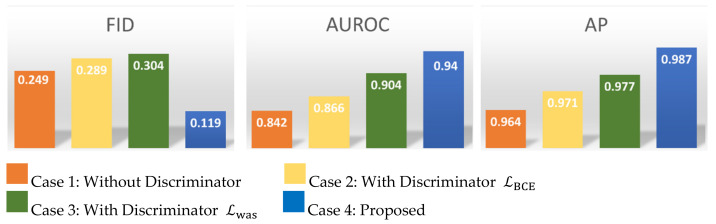
The results of the ablation experiment. Blue is the proposed method, orange is the condition without discriminator, golden is the condition with discriminator $L_{BCE}$, and green is the condition with discriminator $L_{was}$.

Here we demonstrate the utility of the additional components we used to stabilize the adversarial training VAGWDD model by systematically removing each component in turn. It is crucial for us to perform the ablation experiment to display the different combinations of loss functions on the CT-IRT datasets. All proposed loss functions we designed in this paper, shown in Figure 6, can effectively enhance the performance. Specifically, we verify four cases. In the first cases, we consider only $L_{VAE}$, the variational autoencoder structure. In the second and third cases, we use variational autoencoder plus discriminators with the binary cross-entropy loss ($L_{BCE}$) and the Wasserstein distance loss ($L_{Was}$), respectively. In the final case, we consider the full proposed model, VAGWDD. AP and AUROC for 1022 testing images, including the normal and abnormal samples, are displayed in Figure 6. In addition, Fréchet Inception Distance (FID) is a very popular evaluation method for the GAN model recently [45]. We take FID score as the measurement for the difference between the input and reconstructed images.

We notice that FID scores obtained for the proposed model is lower than in other cases. In general, lower scores mean the model regenerated images with higher quality and close to the original ones. The AUROC and AP of case 1 are respectively recoded as 0.842 and 0.964. When the loss function of the discriminator is added by binary cross-entropy ($L_{BCE}$), the performance of the system improves marginally by 2.4% AUROC and 0.7% AP. When replaced by Wasserstein loss function $L_{was}\;$without $L_{WDD-fea}$, the performance improves further by 3.8% and 0.6%. When tested for our proposed structure, performance is further improved by 3.6% and 1%.

## 5. Conclusions

Overheating anomaly detection is an important step in the operation of a power transformer, and accurate fault detection results are of great significance to improve the utilization of power transformers and avoid catastrophic economic losses. we have primarily proposed a deep unsupervised generative adversarial approach to detect anomalous regions within IRT images of cast-resin power transformers. By combining the advantages of variational autoencoder generative adversarial network and Wasserstein distance, the VAG model showed a great reconstruction ability and stability in the training process. In this method, the VAG model is used to generate corresponding images with a normal state. After computing the pixel-wise cosine distance between the original image and the generated image, the faulty features are obtained from the residual image. The comparison experiment results show that our proposed method achieve good performance. The anomaly accuracy, AUROC, F1 scores and AP of the proposed method were significantly higher than those of other anomaly detection models (AE-ssim, AE-L2, GANomaly, RSRAE, ADAE and f-anoGAN). The proposed method also has a smaller number of weight parameters and less storage space. Our next aim is to optimize the proposed algorithms to improve the amount of the parameters and ensure real-time performance in fault detection accuracy. Apart from that, we will explore more effective ways to develop unknown fault detection in unsupervised or semi-supervised learning.

## Figures and Tables

**Table 1:** The number of training and testing images for each category.

Category	Training Dataset	Testing Dataset
F0	300	109
F1	0	126
F2	0	119
F3	0	115
F4	0	124
F5	0	102
F6	0	106
F7	0	117
F8	0	104

**Table 2:** The top10 value of Mean_SSIM and Mean_PSNR of the VAG model training.

Ranking	@Epoch	Mean_SSIM	Mean_PSNR
1	7240	0.99993577	77.668
2	7540	0.99991863	77.291
3	6920	0.99992996	77.679
4	6240	0.99994356	77.328
5	6040	0.99994149	77.570
6	8760	0.99993628	77.812
7	8780	0.99994446	77.998
8	6700	0.99993794	77.684
9	5960	0.99994363	77.647
10	9880	0.99995971	78.807

**Table 3:** The accuracy results of correctly classified anomalous images and normal images for different methods and the proposed method.

Category	AE-ssim	AE-L2	GANomaly	RSRAE	ADAE	Proposed
Only F0	0.743	0.990	0.844	0.596	0.697	0.991
F1 + F0	0.778	0.640	0.278	0.484	0.921	0.960
F2 + F0	0.857	0.695	0.218	0.387	0.891	0.975
F3 + F0	0.513	0.465	0.217	0.383	0.661	0.948
F4 + F0	0.742	0.455	0.218	0.306	0.629	0.944
F5 + F0	0.412	0.535	0.225	0.422	0.559	0.882
F6 + F0	0.406	0.676	0.264	0.377	0.443	0.642
F7 + F0	0.675	0.759	0.197	0.359	0.658	0.940
F8 + F0	0.462	0.573	0.260	0.452	0.567	0.904
mean	0.621	0.643	0.302	0.418	0.670	0.910

**Table 4:** Quantitative comparisons with other methods (AUROC).

Category	AE-ssim	AE-L2	GANomaly	RSRAE	ADAE	f-anoGAN	Proposed
F1 + F0	0.819	0.817	0.532	0.507	0.848	0.468	0.976
F2 + F0	0.834	0.844	0.446	0.428	0.828	0.533	0.983
F3 + F0	0.759	0.730	0.426	0.427	0.716	0.537	0.969
F4 + F0	0.812	0.725	0.452	0.374	0.708	0.591	0.967
F5 + F0	0.727	0.760	0.460	0.466	0.687	0.476	0.937
F6 + F0	0.729	0.835	0.430	0.414	0.594	0.562	0.816
F7 + F0	0.793	0.876	0.455	0.423	0.718	0.553	0.965
F8 + F0	0.741	0.784	0.479	0.465	0.665	0.523	0.947
mean	0.777	0.796	0.460	0.438	0.720	0.530	0.945

**Table 5:** Quantitative comparisons with other method (F1 score).

Category	AE-ssim	AE-L2	GANomaly	RSRAE	ADAE	Proposed
F1 + F0	0.875	0.802	0.217	0.326	0.959	0.974
F2 + F0	0.923	0.836	0.179	0.279	0.942	0.982
F3 + F0	0.678	0.706	0.179	0.277	0.796	0.969
F4 + F0	0.852	0.693	0.179	0.235	0.772	0.966
F5 + F0	0.583	0.751	0.184	0.296	0.717	0.938
F6 + F0	0.577	0.833	0.209	0.274	0.614	0.812
F7 + F0	0.806	0.871	0.164	0.264	0.794	0.965
F8 + F0	0.632	0.777	0.206	0.311	0.724	0.948
mean	0.741	0.784	0.190	0.283	0.790	0.944

**Table 6:** Quantitative comparisons with other method (Average Precision, AP).

Category	AE-ssim	AE-L2	GANomaly	RSRAE	ADAE	f-anoGAN	Proposed
F1 + F0	0.823	0.827	0.570	0.533	0.800	0.522	0.974
F2 + F0	0.821	0.847	0.498	0.476	0.743	0.569	0.980
F3 + F0	0.736	0.733	0.464	0.480	0.621	0.591	0.966
F4 + F0	0.807	0.739	0.498	0.464	0.665	0.646	0.966
F5 + F0	0.692	0.747	0.462	0.462	0.586	0.514	0.930
F6 + F0	0.681	0.828	0.458	0.444	0.526	0.587	0.809
F7 + F0	0.754	0.876	0.481	0.474	0.628	0.604	0.963
F8 + F0	0.684	0.774	0.473	0.478	0.567	0.563	0.941
mean	0.750	0.796	0.488	0.476	0.642	0.575	0.941

**Table 7:** Quantitative comparisons with other methods (parameter and size).

Method	Parameter	Storage Size
f-anoGAN	24.57 M	18 MB
AE-ssim	1.2 M	8.63 MB
AE-L2	1.2 M	8.63 MB
GANomaly	5.43 M	21.8 MB
ADAE	0.101 M	0.258 MB
Proposed	0.12 M	0.550 MB

**Table 8:** Quantitative comparisons with other distance metrics.

Evaluation Metric	Dataset	Sqeuclidean	Euclidean	Manhattan	Cosine (Proposed)
Accuracy	109 testing normal images	0.982	0.982	0.991	0.991
Accuracy	1022 testing images	0.840	0.823	0.843	0.908
AUROC		0.902	0.893	0.908	0.940
F1 scores		0.902	0.890	0.904	0.946
AP		0.979	0.977	0.980	0.987

## Data Availability

The source codes used to support the findings of this study are available from the first author upon request.
